# Editorial: Autophagy: unveiling the mechanisms and implications in health and disease

**DOI:** 10.3389/fphys.2024.1493710

**Published:** 2024-09-30

**Authors:** Shibin Cheng, Xiaodi Chen, Aihua Liao

**Affiliations:** ^1^ Department of Pediatrics, Brown University, Providence, RI, United States; ^2^ Women and Infants Hospital of Rhode Island, Providence, RI, United States; ^3^ Institute of Reproductive Health, Center for Reproductive Medicine, Tongji Medical College, Huazhong University of Science and Technology, Wuhan, China

**Keywords:** autophagy, Alzheimer’s disease, rotator cuff injuries, bile duct carcinoma, oxidative stress

## 1 Introduction

Autophagy, a vital cellular process responsible for maintaining homeostasis by degrading and recycling cellular components, has emerged as a critical area of research in recent years (Nakashima et al., 2023; Cheng et al., 2022a; Nakashima et al., 2020; Cheng et al., 2022b; Chen et al., 2023). This intricate mechanism plays a dual role in health and disease, safeguarding cellular function while also contributing to the pathology of various conditions. This Research Topic explores the complexities of autophagy, shedding light on its diverse roles across a spectrum of diseases, including chronic obstructive pulmonary disease (COPD), rotator cuff injuries, bile duct carcinoma, and Alzheimer’s disease. The featured studies provide fresh insights into the protective and potentially harmful aspects of autophagy, offering new avenues for therapeutic intervention and advancing our understanding of its role in disease progression.

### 1.1 The interplay between oxidative stress and autophagy in chronic obstructive pulmonary disease

In the intricate web of cellular homeostasis, autophagy and oxidative stress are two fundamental players that maintain equilibrium. COPD is a progressive respiratory condition characterized by persistent inflammation and oxidative stress, leading to irreversible airflow limitation (Guan et al., 2024). In this review, Zhao et al. illuminated the nuanced interplay between these processes and their roles in COPD pathogenesis. Oxidative stress results from an imbalance between reactive oxygen species (ROS) and the body’s ability to detoxify them, leading to cellular damage and inflammation. Autophagy, a cellular process responsible for degrading and recycling damaged organelles and proteins, plays a dual role in COPD. On one hand, autophagy helps mitigate oxidative stress by removing damaged mitochondria, which are significant sources of ROS. On the other hand, chronic oxidative stress can impair autophagic processes, leading to the accumulation of damaged cellular components. This disruption contributes to the progression of COPD by promoting inflammation and cellular senescence. Recent insights into this interplay underscore the potential of targeting autophagy pathways as a therapeutic strategy to mitigate oxidative stress and improve outcomes in COPD patients.

### 1.2 The pathology of oxidative stress-induced autophagy in a chronic rotator cuff enthesis tear

Rotator cuff injuries, particularly chronic enthesis tears, pose significant challenges in orthopedic medicine. Enthesis refers to the site where tendons or ligaments attach to bone, and tears at these sites can lead to debilitating pain and functional impairment. The pathology of these injuries is increasingly recognized to involve oxidative stress and autophagy (Kwong et al., 2019). Oxidative stress in rotator cuff tears results from the excessive production of ROS and impaired antioxidant defenses. This imbalance damages cellular components and contributes to inflammation and tissue degeneration. Autophagy in this context appears to be a double-edged sword. While autophagy can help remove damaged cells and repair tissues, excessive or dysregulated autophagy may exacerbate tissue damage and inflammation. The challenge in treating chronic rotator cuff tears lies in modulating autophagy to promote healing while minimizing oxidative stress-induced damage.

### 1.3 Autophagy impairment in human bile duct carcinoma cells

In this research article, Prasetia et al. investigated the activity of autophagy in intrahepatic cholangiocarcinoma, a malignancy that arises from the epithelial cells of the bile ducts by evaluating the abundance of regulatory proteins of autophagic flux, such as LC3II, p62, and TFEB, a master regulator of autophagy-lysosomal biogenesis. Their results suggested autophagy impairment as a significant factor in the development and progression of this cancer. In support of this, the induction of autophagy by rapamycin significantly inhibited the growth of cultured cholangiocarcinoma cells. Autophagy may function as a tumor-suppressive mechanism by degrading damaged cellular components and preventing the accumulation of mutations. Impaired autophagy in bile duct epithelial cells may lead to the accumulation of damaged organelles and proteins, contributing to tumorigenesis. Targeting the autophagy pathway in bile duct carcinoma presents a promising approach to overcoming drug resistance and improving treatment outcomes.

### 1.4 The comprehensive landscape analysis of autophagy in cancer development and drug resistance

Cancer development and drug resistance are driven by a complex interplay of cellular mechanisms, with autophagy emerging as a critical factor in these processes. Li et al. investigated the role of autophagy in cancer progression and drug resistance, revealing its complex dual function. Autophagy can either suppress or promote tumor growth depending on the context. The study utilized a 45-gene autophagy signature to assess activity in tumors from TCGA and GEO databases, categorizing them into high and low autophagy score subtypes. Tumors with high autophagy scores were linked to poorer prognosis and distinct molecular changes, including shifts in immune and hypoxia-related factors. Conversely, tumors with low autophagy scores showed better prognoses and greater immune cell infiltration, including CD8^+^ T cells and various macrophage subtypes. This suggests that autophagy status impacts the tumor immune landscape, which could influence the effectiveness of immune-based therapies.

Additionally, the study examined autophagy’s role in chemoresistance in breast cancer. It was found that dihydroartemisinin and artesunate could reverse doxorubicin resistance by inducing autophagy, as indicated by increased levels of LC3B and ATG7. This research highlights the intricate role of autophagy in cancer and its potential as a therapeutic target for improving treatment outcomes and overcoming drug resistance.

### 1.5 Proteostasis and neurodegeneration: a closer look at autophagy in Alzheimer's disease

Barmaki et al. reviewed the critical role of autophagy dysfunction in Alzheimer’s disease (AD). Autophagy, a key cellular process for maintaining protein balance and organelle health, is disrupted in AD, leading to the accumulation of amyloid-beta plaques, tau tangles, and damaged organelles—core features of the disease.

The review emphasizes the complex interactions between autophagy and other cellular systems, such as mitochondrial function and endoplasmic reticulum stress. Dysfunction in these interconnected pathways further exacerbates the progression of AD. For instance, impaired autophagy can contribute to mitochondrial dysfunction, increasing oxidative stress and cellular damage. Effective AD therapies may need to address these related systems alongside autophagy. Genetic and environmental factors influencing autophagy also impact AD susceptibility. Variations in genes encoding autophagic proteins have been linked to an increased risk of the disease, suggesting that understanding these genetic factors could help in identifying at-risk individuals and tailoring preventative strategies.

Recent advances in autophagy-modulating drugs show potential for AD treatment by enhancing protein clearance and reducing neurotoxic aggregates in preclinical models. However, translating these findings into clinical practice requires careful evaluation of drug dosing, safety, and long-term efficacy. The study advocates for continued research and clinical trials to optimize autophagy-based therapies for AD.

Overall, targeting autophagy mechanisms holds promise for developing new therapeutic strategies to slow or potentially halt the progression of Alzheimer’s disease.

## 2 Conclusion

The papers in this Research Topic explore the critical role of autophagy in COPD, rotator cuff injuries, bile duct carcinoma, and Alzheimer’s disease, highlighting its dual nature as both a protector and potential contributor to disease. While these studies provide valuable insights, they represent just a fraction of the broader spectrum of conditions in which autophagy dysregulation plays a key role, including adverse pregnancy outcomes, renal and cardiovascular diseases, diabetes, and obesity (Nakashima et al., 2023; Cheng et al., 2022a; Nakashima et al., 2020; Cheng et al., 2022b; Chen et al., 2023). The intricate balance of autophagic activity is essential for maintaining health. As we gain a deeper understanding of autophagy’s multifaceted roles, new therapeutic avenues are emerging, offering hope for more effective treatments across various medical fields. However, the real challenge lies in precisely modulating autophagy to maximize its benefits while avoiding the risk of exacerbating disease. This underscores the need for continued research and innovation in this dynamic and rapidly evolving area of study.
